# Genetic and Environmental Influences on Sexual Orientation: Moderation by Childhood Gender Nonconformity and Early-Life Adversity

**DOI:** 10.1007/s10508-023-02761-w

**Published:** 2023-12-28

**Authors:** Olakunle Ayokunmi Oginni, Katarina Alanko, Patrick Jern, Frühling Vesta Rijsdijk

**Affiliations:** 1 The Social, Genetic and Developmental Psychiatry Centre, Institute of Psychiatry, Psychology and Neuroscience, Denmark Hill, King’s College London, London, SE5 8AF UK; 2 Department of Mental Health, Obafemi Awolowo University Teaching Hospitals Complex, Ile-Ife, Nigeria; 3 Division of Psychological Medicine and Clinical Neuroscience, Wolfson Centre for Young People’s Mental Health, Cardiff University, Cardiff, UK; 4 Department of Psychology, Åbo Akademi University, Turku, Finland; 5 Department of Psychology, Faculty of Social Sciences, Anton de Kom University, Paramaribo, Suriname

**Keywords:** Sexual orientation, Childhood gender nonconformity, Early-life adversity, Moderation, Twins

## Abstract

Existing evidence indicates genetic and non-genetic influences on sexual orientation; however, the possibility of gene-environment interplay has not been previously formally tested despite theories indicating this. Using a Finnish twin cohort, this study investigated whether childhood gender nonconformity and early-life adversities independently moderated individual differences in sexual orientation and childhood gender nonconformity, the relationship between them, and the etiological bases of the proposed moderation effects. Sexual orientation, childhood gender nonconformity, and early-life adversities were assessed using standard questionnaires. Structural equation twin model fitting was carried out using OpenMx. Childhood gender nonconformity was significantly associated with reduced phenotypic variance in sexual orientation (*β* =  − 0.14, 95% CI − 0.27, − 0.01). A breakdown of the underlying influences of this moderation effect showed that this was mostly due to moderation of individual-specific environmental influences which significantly decreased as childhood gender nonconformity increased (*β*_E_ =  − 0.38; 95% CI − 0.52, − 0.001) while additive genetic influences were not significantly moderated (*β*_A_ = 0.05; 95% CI − 0.30, 0.27). We also observed that the relationship between sexual orientation and childhood gender nonconformity was stronger at higher levels of childhood gender nonconformity (*β* = 0.10, 95% CI 0.05, 0.14); however, significance of the underlying genetic and environmental influences on this relationship could not be established in this sample. The findings indicate that beyond a correlation of their genetic and individual-specific environmental influences, childhood gender nonconformity is further significantly associated with reduced individual-specific influences on sexual orientation.

**Supplementary Information:**

The online version contains supplementary material available at 10.1007/s10508-023-02761-w.

## Introduction

### Genetic Influences on Sexual Orientation

From a behavioral genetics perspective, attempts to understand the origins of sexual orientation (i.e. sex-specific romantic and erotic attraction) can be categorized into those focused on genetic factors, environmental factors and combinations of these, respectively. Evidence for genetic factors includes familial clustering of non-heterosexuality (Bailey & Bell, 1993). Similarly, twin studies have estimated the variance in sexual orientation due to genetic factors (heritability) as ranging between 30 and 50% (Alanko et al., 2010; Kendler et al., 2000; Långström et al., 2010) with the residual variance being explained by individual-specific environmental factors. Attempts to identify specific genetic loci have yielded inconsistent findings (Bailey et al., 2016). Initially, these tended to investigate individual gene effects; however, the current understanding of the genetic architecture of complex traits indicates that most behavioral traits are polygenic, i.e., influenced by thousands of genes of small individual effects (Manolio et al., 2009). An earlier genome-wide association study—GWAS (Sanders et al., 2017) did not identify common genetic variants (single nucleotide polymorphisms—SNPs) that achieved genome-wide significant association with sexual orientation. More recently, a much larger GWAS identified five genetic loci associated with same-sex sexual behavior (Ganna et al., 2019). Ganna et al. further estimated the SNP-based heritability to be 8–25% which is lower than the range of estimates from twin-based approaches. Among other factors, this difference in heritability estimates from SNP-based and family-based methods (the “missing” heritability) is typically attributed to the small effect size of the individual genetic variants, the exclusion of rare and structural variants, and the possibility of gene-environment interactions (Dick, 2011; Manolio et al., 2009). These limitations may be, respectively, overcome by larger sample sizes and whole genome-sequencing approaches, and we discuss the relevance of gene-environment interactions to the development of sexual orientation after reviewing environmental influences.

### Environmental Influences on Sexual Orientation

The environment in this context refers to all non-genetic etiological influences on sexual orientation. Initial theories about non-genetic influences on sexual orientation were based on social learning theories, stating that non-heterosexual individuals are “recruited” by existing non-heterosexual individuals, or that they learn non-heterosexual behavior from non-heterosexual parents or relatives. However, these have not been supported by empirical evidence (Bailey et al., 2016). More recently, research on environmental influences have focused on the effects of gonadal hormones at critical periods such as during prenatal development. These research efforts propose that exposure to gonadal hormones at a critical period during in-utero development influences gendered behavior and later sexual orientation (Bao & Swaab, 2011). This is supported by findings from animal studies in which exposure to cross-sex gonadal hormones during prenatal development is associated with cross-sex sexual behavior in adulthood (Södersten, 2015). While animal studies are not completely generalizable to human subjects, the potential role of prenatal hormones in human sexual orientation is supported by findings suggesting that non-heterosexual brains are shifted toward the opposite sex in terms of anatomy and physiology (Bao & Swaab, 2011; Rahman, 2005). Prenatal gonadal hormones are thus thought to influence the organization of the brain in a sex-specific direction (Bailey et al., 2016). Some studies have investigated proxy measures of prenatal gonadal hormone exposure such as digit-length ratios, however, the findings have been mixed (McFadden et al., 2005).

### Childhood Gender Nonconformity and Early-Life Adversities as Moderators of the Etiological Influences on Sexual Orientation

It is increasingly recognized that genetic and environmental factors are seldom completely independent of one another (Dick, 2011). A relevant form of gene-environment interplay that has not been systematically investigated in the etiology of sexual orientation is gene-environment interactions. These describe the moderation of genetic influences on a trait by environmental factors, that is, the regulation of sensitivity to genetic influences by environmental exposures (Dick, 2011). These mean that genetic propensities for a trait differentially manifest or are triggered in certain environmental contexts. Specifically, it is possible that the etiological influences on sexual orientation are stronger in specific contexts. This possibility is suggested by the empirical evidence on nonlinear links between childhood gender nonconformity (Li et al., 2017) and Rind’s (2013) counter-normative theory.

Childhood gender nonconformity (CGN) is one of the most consistent predictors of non-heterosexuality (Li et al., 2017; Rieger et al., 2008) and consistent with the principles of behavioral genetics, this observed association can be resolved into genetic and environmental components (i.e., genetic and environmental correlations between both variables). These correlations, thus, imply that the genetic and environmental influences on sexual orientation include those that are unique to it and those shared with CGN (Alanko et al., 2010; Bailey et al., 2000; Burri et al., 2015). Moderator effects of CGN are suggested by evidence that the association between CGN and SO (Rieger et al., 2008; Watts et al., 2018) varies with the level of CGN. For example, using longitudinal data, Li et al. (2017) showed that non-heterosexual adolescents were already more gender nonconforming compared to heterosexual adolescents at 2.5 years of age. Furthermore, this difference significantly increased over time such that two years later, children who went on to identify as heterosexual adolescents were more gender conforming while those who went on to identify as non-heterosexual adolescents became more gender nonconforming. This increasing gender nonconformity among children who went on to identify as non-heterosexual suggests a nonlinear relationship between CGN and non-heterosexuality during development whereby CGN during childhood was associated with a further increased likelihood of later non-heterosexuality, i.e., gender nonconformity moderating its relationship with sexual orientation (Keith, 2015). Considering that every observed phenotypic association can be resolved into genetic and environmental components, including that between sexual orientation and CGN; the finding by Li et al., (2017) raises the possibility suggests that the genetic and environmental influences underlying the association between sexual orientation and CGN (i.e., genetic and environmental influences on sexual orientation shared with CGN) may be moderated by CGN. This moderation effect may be explained as CGN indicating an increased sensitivity to the genetic (and/or environmental) influences on sexual orientation.

With respect to moderation by ELA, Rind (2013) suggested that “counter-normative” childhood family environments characterized by adversities may “…elicit homosexual expression…especially in those biologically pre-inclined…”. A biological pre-inclination indicates genetic predisposition and “homosexual expression” would refer to the manifestation of a genetic predisposition to same-sex sexuality. The elicitation of the expression of the genetic predisposition to same-sex sexuality by “counter-normative” family environments is indicative of a gene-environment interaction whereby the genetic propensity for same-sex sexual orientation is unmasked by adverse family circumstances. This possibility is consistent with previously reported associations between same-sex sexuality and early-life adversities (e.g., Andersen & Blosnich, 2013; Baams, 2018) and was proposed as a counter-explanation to the suggestion that early-life adversities are causal for same-sex attraction (Roberts et al., 2013). In other words, Rind’s theory suggests that early family circumstances may increase the sensitivity to etiological influences on sexual orientation. According to Rind (2013), such circumstances include parental alcohol problems and mental illness and unwanted childhood sexual experience which along with physical and emotional abuse and neglect constitute childhood or early-life adversities (Andersen & Blosnich, 2013). Rind’s theory thus raises the possibility that the small but significant associations between early-life adversities (ELA) and sexual orientation (Andersen & Blosnich, 2013; Baams, 2018; Roberts et al., 2013; Xu et al., 2020) may mask nonlinear moderation effects. As some of the genetic and environmental influences on SO are shared with CGN (Alanko et al., 2010; Bailey & Zucker, 1995) the genetic and environmental correlations between SO and CGN may also be positively moderated by ELA.

Although the above theories suggest moderation of mean effects, investigating the moderation of individual differences can further demonstrate the impact of moderator variables whereby negative moderation of phenotypic variance (i.e., decreasing variance with increasing levels of the moderator) indicates greater importance of the moderator variable (indexed by the reduced variance of or “noise” around the outcome variable at higher levels of the predictor/moderator variable; Downs & Rocke, 1979). This contrasts with an increase in the variance of an outcome variable at higher levels of the predictor (or moderator) variable. By resolving phenotypic variance into latent genetic and environmental components, the classical twin design can further determine the extents to which observed phenotypic moderation is based on moderation of underlying genetic and environmental components (Purcell, 2002).

Frameworks for gene-environment interactions include diathesis-stress (genetic influences manifesting negatively in adverse environments; Monroe & Simons, 1991), vantage sensitivity (genetic influences manifest in favorable environments; Pluess & Belsky, 2013) or differential susceptibility (genetic influences manifesting as positive or negative in favorable or adverse environments, respectively; Belsky & Pluess, 2009). These often imply a purpose, i.e., positive or negative (Manuck & McCaffery, 2014) which may not be applicable to sexual orientation. However, it is also possible that same-sex sexuality is part of an evolutionarily adaptive response to adversities (Rind, 2013).

A better understanding of the etiological influences on sexual orientation could indirectly facilitate the wellbeing of non-heterosexual individuals by reducing discrimination and improving self-acceptance (Bailey et al., 2016; Hammack-Aviran et al., 2022). Furthermore, the mental health disparities in non-heterosexual individuals which are partly attributable to minority stress (Meyer, 2003) and possible genetic relationships (Frisell et al., 2010; Ganna et al., 2019; Zietsch et al., 2012) may also be partly explained by the proposed moderators (CGN and ELA, Jones et al., 2017; McLaughlin et al., 2012; Oginni et al., 2022). Thus, a clearer understanding of the relationship between SO and CGN and ELA may help to better understand the mechanisms of the mental health disparities in non-heterosexual individuals.

Considering that etiological influences on sexual orientation include those unique to sexual orientation and those shared with childhood gender nonconformity (Alanko et al., 2010), the objectives of this study were to investigate whether childhood gender nonconformity and early-life adversities individually moderate (1) the individual differences in sexual orientation and childhood gender nonconformity; (2) the relationship between childhood gender nonconformity and sexual orientation and (3) the genetic and environmental influences on the effects in (1) and (2) We did not have specific hypotheses about moderation of the individual differences in sexual orientation by CGN or ELA; however, in line with Rind’s counter-normativity theory, we expected that genetic influences on individual differences in sexual orientation will be larger at higher levels of ELA. Based on prior research (Li et al., 2017), we hypothesized that the relationship between sexual orientation and childhood gender nonconformity will be moderated by childhood gender nonconformity and that this moderation would be driven by individual-specific environmental influences. Based on Rind’s counter-normativity theory, we expected that the relationship between sexual orientation and childhood gender nonconformity will be stronger at higher levels of ELA and that this moderation would be driven by genetic influences.

## Method

### Participants

The study sample was derived from the Finnish Genetics of Sexuality and Aggression twin cohort. The sample was collected in 2005 (for further information, please see Johansson et al., 2013). Monozygotic and dizygotic twin pairs were identified from the Central Population Registry of Finland and the current study is based on participants in the first wave of data collection. Questionnaires were mailed to 5,000 twin pairs, of which 3604 individuals responded, yielding a response rate of 36% which is typical of mail surveys (Guo et al., 2016) and consistent with response rates from sexuality-related research (Barth et al., 2016; Elliott et al., 2015). This may reflect the sensitive nature of the questions asked about sexuality-related themes (Edwards et al., 2009), although the responses were anonymized to ensure confidentiality. One hundred and forty-two participants were excluded due to incomplete responses and a further 287 due to indeterminate zygosities, leaving a total of 3175 individuals on which phenotypic analyses were based. Of these, there were 872 complete twin pairs (319 monozygotic and 553 dizygotic twins), and subsequent genetic moderation analyses were based on these. Incomplete twin pairs were, however, included to facilitate the estimation of the variances and covariances. The ascertainment of zygosity has been previously described (Christiansen et al., 2003).

### Measures

#### Sexual Orientation

This was assessed by four questions taken from the 12-item Sell assessment of sexual orientation (Sell, 1996). The selected questions focused on sexual attraction toward and sexual behavior with same-sex persons as follows: “How many different men (women for female participants) have you been sexually attracted to in the past year?”; “With how many different men have you engaged in sexual activity in the past year?”; “How often have you, on average, felt sexual attraction toward men in the past year?”; and “How often have you, on average, engaged in sexual activity with a man in the past year?”. The first two questions were scored on an 8-point scale ranging from “None” (0) to “100 or more” (7), while the latter two questions were scored on a 7-point scale ranging from “Never” (0) to “Every day” (6). The mean of the scores on all four questions was derived and the Cronbach’s alphas for the items in this study were 0.89, respectively. However, based on the skewed distribution, the variable was dichotomized with those scoring “0” categorized as heterosexual and those scoring greater than 0 categorized as non-heterosexual.

#### Childhood Gender Nonconformity

This was assessed using a shortened version of the Recalled Childhood Gender Identity/Gender Role Questionnaire (Alanko et al., 2010; Zucker et al., 2006). This version consisted of 13 items assessing gendered behavior of the participants before the age of 12 years and each item was rated on a 5-point Likert scale ranging from 1 to 5. The responses were coded such that higher values were indicative of gender-nonconforming behavior. A response was included for some of the items to indicate that the behaviors described did not apply to them and these were not included in the calculation of the total score. The total score was derived by multiplying the mean of the responses by the number of items, and this score was used in subsequent analyses. The Cronbach’s alpha for the items in this study was 0.85 in male and female participants, respectively.

#### Early-Life Adversities

These were measured using the 28-item Childhood Trauma Questionnaire which assesses emotional, physical and sexual abuse, and emotional and physical neglect occurring before the age of 18 years (Bernstein & Fink, 1998). Three items which constituted a minimization or denial scale to assess the accuracy of the information provided were excluded from the total score. The items were scored on a 5-point Likert scale ranging from 1 (Never True) to 5 (Very Often True). The responses to the items were summed and the total scores used in subsequent analyses. Higher scores indicated increasing early-life adversities. The Cronbach’s alpha in this study was 0.90.

The moderator variables were childhood gender nonconformity and early-life adversities while the moderated parameters were the variances and covariance of sexual orientation and childhood gender nonconformity. The covariates were age, which was assessed in years using a single question; and (birth) sex which was ascertained from the Finnish Central Population Registry.

### Statistical Analyses

#### Data Preparation and Summary Statistics

Model-fitting analyses were carried out using OpenMx in R (Boker et al., 2011). Since the program cannot handle missing definition variables (i.e., variables that moderate path estimates), imputation was carried out to maximize the number of complete twin pairs with non-missing moderator data. Participants with greater than 75% missing responses for any of the three variables were excluded from analyses. Expectation–maximization was selected as the imputation method due to its compatibility with maximum likelihood estimation methods (Dong & Peng, 2013) and SPSS version 25 (IBM Corp, 2017) was used. Sexual orientation was specified as a liability threshold variable with the assumption that its categories reflect an underlying normal distributed liability (Rijsdijk & Sham, 2002). As is typically done in twin studies (McGue & Bouchard, 1984), we adjusted for age and birth sex by regressing them out of the continuous variables and using the log-transformed residuals in subsequent analyses while including both variables in the threshold model for sexual orientation.

#### Structural Equation Modeling on Twin Data

Maximum likelihood estimation was used to estimate the polychoric correlations between sexual orientation, childhood gender nonconformity and early-life adversities. Consistent with the assumptions of genetic models, within-person correlations were constrained to be equal across birth order and zygosity. Symmetric cross-twin correlations were also compared across monozygotic same-sex and dizygotic same- and opposite-sex twin pairs to indicate the relative importance of genetic and environmental factors in the variance–covariance relationships of the variables of interest.

#### Twin Model Fitting

The twin model resolves variance into additive genetic (*A*), shared environmental (*C*) and unique or individual-specific environmental (*E*) components by comparing within-twin pair correlations in monozygotic and dizygotic twins; path coefficients are, respectively, indicated by *a*, *c* and *e*. This is made possible by the underlying assumption that twins reared together share the family environment to the same extent. Thus, the *C* component correlation within monozygotic and dizygotic twin pairs is held to be 1. Furthermore, based on evidence from biometric genetics, monozygotic twins are held to be genetically identical, while dizygotic twins are on 50% genetically identical (Rijsdijk & Sham, 2002). Thus, the *A* correlations within monozygotic and dizygotic twin pairs are 1 and 0.5, respectively. Individual-specific environmental components indicate environmental influences that make individuals different from one another and incorporate measurement error. Hence, *E* correlations across twin pairs are held to be equal to 0. Preliminary model-fitting was therefore carried out to estimate the influence of the *ACE* variance components on the variance–covariance relationships of the variables of interest.

#### Phenotypic Moderation Analyses

Moderation by CGN and ELA of the phenotypic variance–covariance relationships between CGN and SO were investigated in two separate models using the Cholesky decomposition. In the first model, CGN was specified to moderate the covariance between CGN and SO and the variance of SO. In the second model, ELA was specified to moderate the CGN-SO covariance as well as the variances for both CGN and SO. In this second model, in order not to confound the interaction effects, the main effects of ELA on the dependent variables (CGN and SO) were regressed out. In both models, means were constrained to be equal across birth order and zygosity and within-person variances were constrained to be equal across zygosity.

#### Genetic Moderation Analyses

**Fig. 1:**
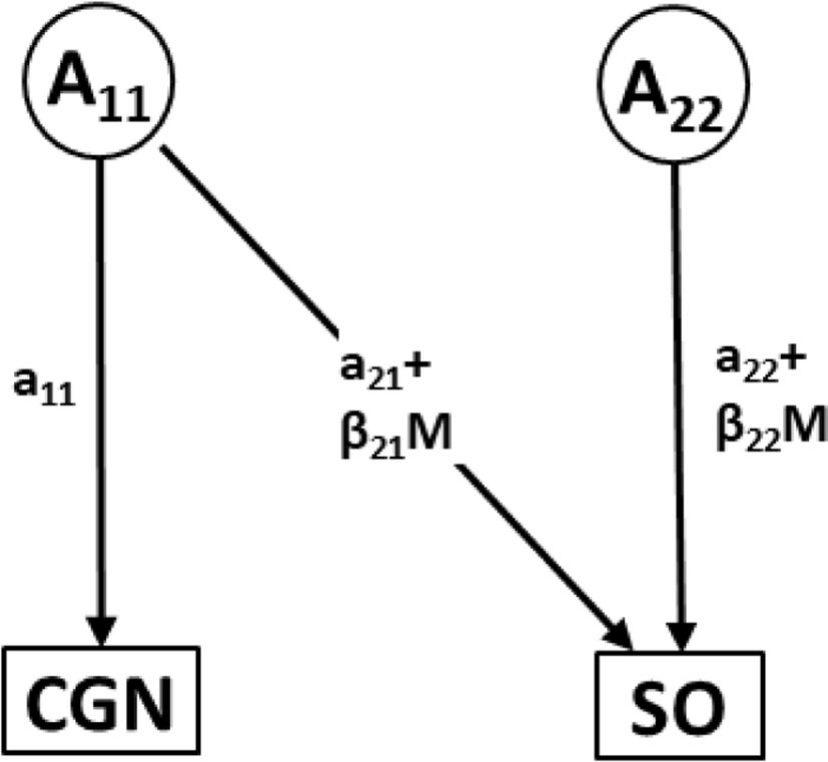
Bivariate moderation model with the moderator (childhood gender nonconformity—CGN) specified as a dependent variable. In this model, CGN is modeled as a dependent variable, allowing for its covariance with sexual orientation (SO) to be moderated by itself. *A*_11_ and *A*_22_ are unique additive genetic influences on CGN and SO, respectively; a_11_ and a_22_ denote their respective unmoderated coefficients, *a*_21_ denotes the additive genetic coefficient of covariance between CGN and SO; (*β*_22_ + *M*) and (*β*_21_ + *M*) denote the respective moderation terms on the paths where M indicates the possible values of the moderator—CGN. Only Additive genetic (*A*) effects are depicted in this figure to facilitate clarity, similar parameters are specified for shared (*C*) and non-shared environmental (*E*) influences

These were carried out using the model described by Purcell (2002). In the first model, CGN was included both as the moderator and as a dependent variable while SO was included as a dependent variable. This bivariate approach allowed for the investigation of the moderation of SO variance components and moderation of the covariance between SO and CGN. In this model, moderated coefficients (*β*_A_, *β*_C_ and *β*_E_) were specified for the *A*, *C* and *E* variance and covariance components in addition to unmoderated terms (*a*, *c* and *e* respectively, see Fig. 1). Thus, the total path coefficient for each of the variance components was derived as a sum of the unmoderated and moderated portions as follows; *a* + *β*_A_*M*, *c* + *β*_C_*M* and *e* + *β*_E_*M* for *A*, *C* and *E* components, respectively, where *M* is any value of the moderator.

**Fig. 2:**
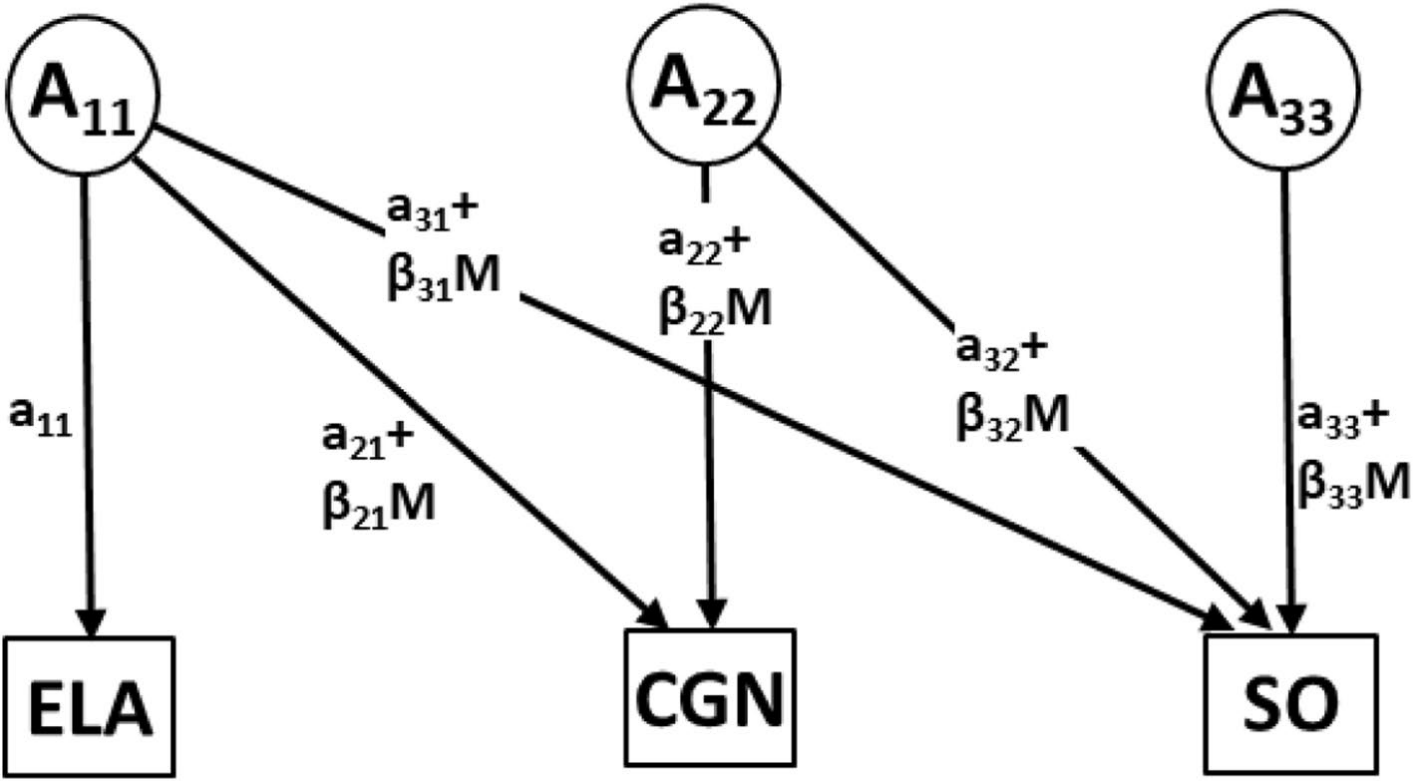
Trivariate moderation model with the moderator (early-life adversities—ELA) modeled as a dependent variable. In this model, ELA is modeled as a dependent variable which allows for its covariance with childhood gender nonconformity (CGN) and sexual orientation (SO) to be moderated by itself. *A*_11_, *A*_22_ and *A*_33_ are unique additive genetic influences on ELA, CGN, and SO, respectively; *a*_11_, *a*_22_ and *a*_33_ denote their respective unmoderated coefficients, *a*_21_, *a*_31_ and *a*_32_ denote the additive genetic coefficients of covariances between ELA and CGN and SO, and between CGN and SO, respectively; (*β*_22_ + *M*), (*β*_33_ + *M*), (*β*_21_ + *M*), (*β*_31_ + *M*) and (*β*_32_ + *M*) denote the respective moderated terms on the paths where M indicates the possible values of the moderator—ELA. Only Additive genetic (*A*) effects are depicted in this figure to facilitate clarity, similar parameters are specified for shared (*C*) and non-shared environmental (*E*) influences. The arrangement of the variables is based on ease of analyses rather than any causal inferences and the interpretation of the covariance paths is correlational rather than causal

In the second model, early-life adversity (ELA) was included as the moderator. For these analyses, we extended the previously described bivariate model to include three dependent variables—ELA (the moderator modeled as a dependent variable), CGN and SO (see Fig. 2). An alternative approach would be to model CGN and SO as the only dependent variables while ELA would be included only as the moderator with its main effects included in the mean terms of both CGN and SO. However, an advantage of this trivariate approach is that it allows for the investigation of moderation of the covariance between ELA and the other dependent variables. Only moderation of the CGN-SO variance–covariance relationships by ELA are reported in the main results because these are the focus of the current study. We wish to emphasize that the focus of the present study is moderation of the variance components and that the interpretation of the covariances of ELA with CGN and SO is correlational (Loehlin, 1996) rather than causal. As both ELA and CGN were assessed retrospectively, the present sample cannot indicate the temporal order of occurrence. Similarly, although CGN and ELA are often presumed to have occurred earlier than the recognition of sexual orientation (Li et al., 2017; Xu et al., 2020), it is possible that the developmental processes involved in the determination of sexual orientation had occurred earlier and simultaneously with the manifestation of CGN and/or the occurrence of ELA.

The significance of the moderation terms was determined by the 95% maximum likelihood confidence intervals, which are consistent with the likelihood ratio tests of sub-models excluding these effects. Intervals containing 0 indicate non-significance. To simplify the results, the *C* parameters were dropped from the moderation models because the standardized *C* parameter influences on the covariance between childhood gender nonconformity and sexual orientation were mostly null (unstandardized variance components ranged between − 0.01 and 0.00; *χ*^2^[6] = 0.56, *p* = 1.00). As recommended (Purcell, 2002), we plotted the unstandardized moderation terms as functions of the values of the moderator (ranging between the mean ± 3SD; see supplementary material).

## Results

### Descriptive Statistics

The mean age of the participants was 37.6 (± 2.94) years (Table 1) and the mean scores for early-life adversity and childhood gender nonconformity were 37.6 (± 12.30) and 26.4 (± 8.08), respectively. Regarding sexual orientation, 6.8% were categorized as non-heterosexual and this was comparable in male (6.5%) and female participants (7.0%; *χ*^2^ = 0.28, *p* = 0.60).

**Table 1:** Descriptive statistics of the observed variables per sex-zygosity groups

	*n*	Age Mean (SD)	ELA Mean (SD)	CGN Mean (SD)	SO (non-heterosexual) *n* (%)
Total	3175	37.5 (2.92)	37.4 (12.22)	26.3 (8.12)	216 (6.80)
MZ	1054	37.8 (2.92)	37.3 (12.18)	26.4 (8.31)	66 (6.26)
DZ	2121	37.4 (2.91)	37.5 (12.25)	26.3 (8.03)	150 (7.07)

The scale ranges for ELA and CGN are 25–125, 13–65 respectively

*ELA* Early-life adversities, *CGN* Childhood gender nonconformity, *SO* Sexual orientation, *MZM* Monozygotic same-sex male twins, *DZM* Dizygotic same-sex male twins, *MZF* Monozygotic same-sex female twins, *DZF* Dizygotic same-sex female twins, *DZO* Dizygotic opposite-sex twins, *SD* Standard deviation

### Phenotypic Correlations

There were significant positive within-person correlations between early-life adversities, childhood gender nonconformity and sexual orientation (Table 2), however, the association between early-life adversities and sexual orientation was attenuated when CGN was included in the model (*β* =  − 0.29, 95% CI − 0.65, 0.03). The cross-twin within-variable correlations were significantly larger in monozygotic twins relative to dizygotic twins for all three variables. This indicated the contribution of genetic factors to the etiology of all three variables. However, the within-trait cross-twin correlations were less than 1 for all variables, indicating significant individual-specific environmental influences and measurement error.

**Table 2:** Within-person and cross-twin correlations of study variables with 95% confidence intervals

	ELA (3)	CGN (2)	SO (1)
*Within-person*			
(1)	1.00		
(2)	0.15 (0.12, 0.19)	1.00	
(3)	0.23 (0.16, 0.29)	0.27 (0.21, 0.33)	1.00
*Cross-twin monozygotic*			
(1)	0.63 (0.56, 0.68)		
(2)	0.10 (0.05, 0.16)	0.48 (0.41, 0.55)	
(3)	0.17 (0.06, 0.28)	0.22 (0.11, 0.33)	0.56 (0.26, 0.77)
*Dizygotic*			
(1)	0.34 (0.26, 0.41)		
(2)	0.07 (0.01, 0.12)	0.03 (− 0.05, 0.12)	
(3)	0.08 (− 0.03, 0.18)	− 0.04 (− 0.15, 0.08)	0.15 (− 0.15, 0.42)

*ELA* Early-life adversities, *CGN* Childhood gender nonconformity, *SO* Sexual orientation

### Multivariate Model Fitting

Preliminary multivariate twin model-fitting indicated significant influences of additive genetic and individual-specific environmental factors in the etiology of all three variables (Table 3). Shared environmental influences were close to nought and not statistically significant for all three variables.

**Table 3:** Standardized variance components and 95% confidence intervals for observed variables

	*h*^2^			*c*^2^			*e*^2^		
	ELA	CGN	SO	ELA	CGN	SO	ELA	CGN	SO
ELA	0.57 (0.39, 0.68)			0.05 (0.00, 0.20)			0.37 (0.32, 0.44)		
CGN	0.64 (0.06, 5.3E2)	0.40 (0.31, 0.47)		0.09 (− 0.21, 0.51)	0.00 (0.00, 0.05)		0.27 (− 0.02, 0.42)	0.60 (0.52, 0.67)	
SO	0.85 (− 9.1E2, 9.8E4)	0.64 (− 2.3E3, 0.82)	0.51 (0.02, 0.73)	− 0.07 (− 3.3E6, 0.77)	− 0.01 (− 1.8E3, 0.24)	0.00 (0.00, 0.42)	0.21 (− 76.46, 3.0E3)	0.38 (− 0.03, 1.9E3)	0.49 (0.27, 0.77)

The diagonals of each matrix give the proportion of variable variance determined by the respective variance component, while the off-diagonals represent the proportion of covariance determined

Degrees of freedom = 9503, −  2LL = 19,055.77, AIC =  49.77

*h*^2^, *c*^2^ and *e*^2^ Proportions contributed by additive genetic, shared and unique environmental factors respectively, *ELA* Early-life adversities, *CGN* Childhood gender nonconformity, *SO* Sexual orientation

### Phenotypic Moderation

**Fig. 3:**
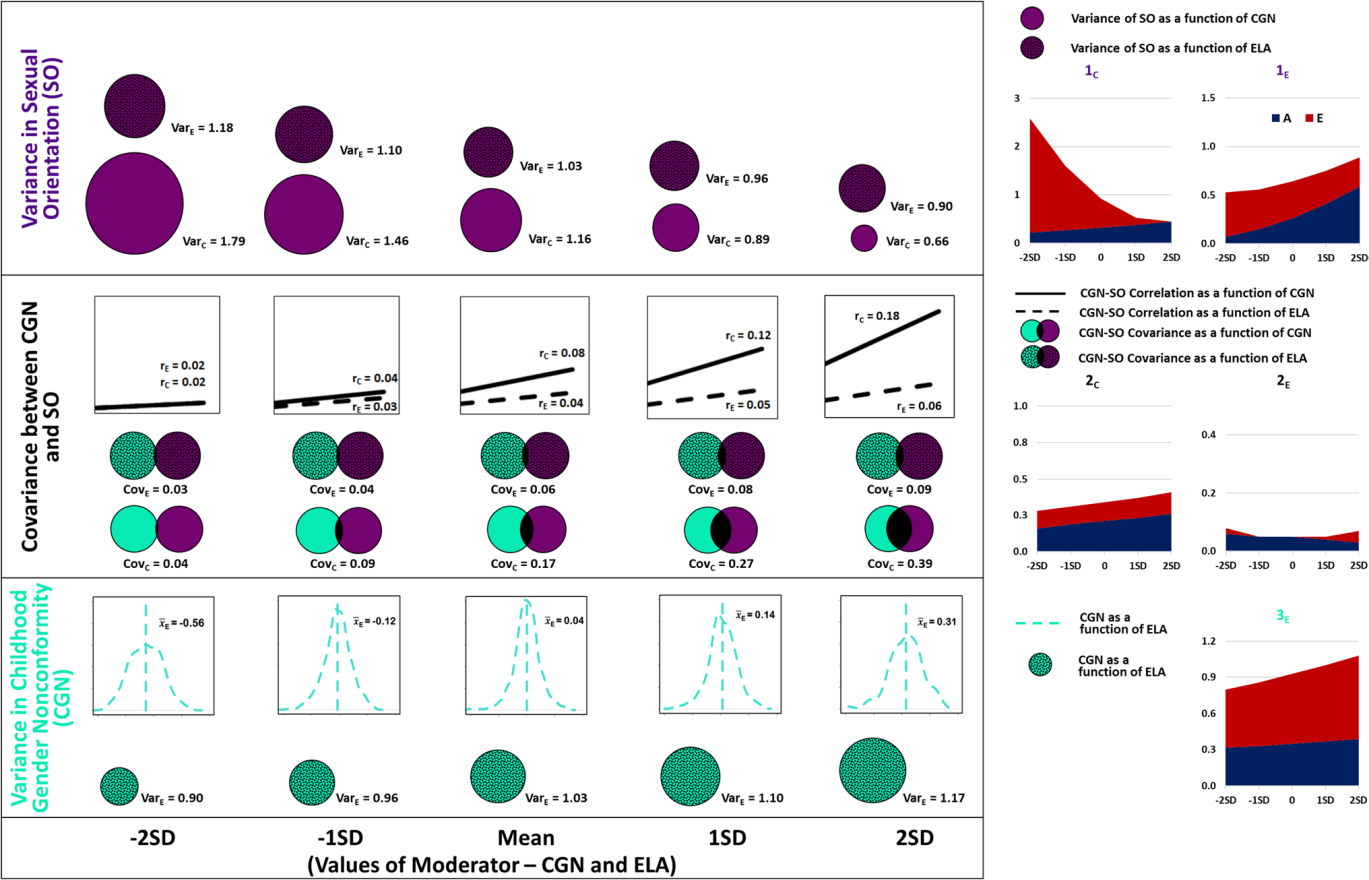
Plots showing changes in the means and variances of Childhood Gender Nonconformity (CGN) and Sexual Orientation (SO) and their covariance and correlation across different levels (mean ± 2 standard deviation units) of the moderators—CGN and Early-Life Adversities (ELA). *Note*: Plots 1, 3, and 2 indicate Genetic (*A*) and Unique Environmental (*E*) influences on the variances of Sexual orientation and CGN, and their covariance, respectively, Subscripts _C_ and _E_ indicate the specific moderator effect being depicted—CGN and ELA respectively, SD indicates Standard deviation, $\overline{x}$ indicates the mean (± standard deviation), *Var* Variance, *r* Correlation coefficient, *Cov* Covariance. The scales for the plots depicting moderation of *A* and *E* influences on SO variance and its covariance with CGN by ELA are halved to allow comparison with plots depicting moderation by CGN while facilitating visibility

In the first phenotypic moderation model (CGN as the moderator), both the CGN-SO covariance and the SO variance paths were significantly moderated by CGN positively and negatively, respectively, (Table 4) such that the CGN-SO covariance was maximal while SO variance was lowest at the highest levels of CGN (Fig. 3; Fig. S1a, b in supplementary material). In the second model (ELA as the moderator), however, only the variance of CGN was significantly moderated whereby the variance of CGN was highest at the highest levels of ELA.

**Table 4:** Unstandardized estimates and 95% confidence intervals for the moderation parameters of the phenotypic variance–covariance relationships between childhood gender nonconformity and sexual orientation

Moderator	CGN variance	CGN-SO covariance	SO variance
	Estimate (95% CI)	Estimate (95% CI)	Estimate (95% CI)
CGN (Model 1)^a^		**0.10 (0.05, 0.14)**	− **0.13 (**− **0.27,** − **0.01)**
ELA (Model 2)^b^	**0.03 (0.01, 0.06)**	0.03 (− 0.05, 0.10)	− 0.04 (− 0.26, 0.13)

Bold parameters indicate statistically signficant parameter estimates

^a^Degrees of freedom = 6412, − 2LL = 10,653.16, AIC =  − 2170.84

^b^Degrees of freedom = 6409, − 2LL = 10,582.64, AIC =  − 2235.36

### Genetic Moderation

In the first genetic moderation model (CGN as the moderator), ***A*** and ***E*** influences on the relationship between CGN and SO as CGN did not significantly vary with CGN (*β*_A_ = 0.03; 95% CI − 0.14, 0.20; *β*_E_ = 0.01; 95% CI − 0.18, 0.17, Table 5, Fig. 3). However, *E* influences on the variance of sexual orientation significantly decreased as CGN increased (*β*_E_ =  − 0.38; 95% CI − 0.52, − 0.001, Table 5, Fig. 3) such that at the highest levels of CGN, *E* influences were completely attenuated whereas ***A*** influences on SO variance did not significantly vary by CGN (*β*_A_ = 0.05; 95% CI − 0.30, 0.27).

**Table 5:** Unstandardized estimates and 95% confidence intervals for the moderation parameters of the component influences on variance–covariance relationships between childhood gender nonconformity and sexual orientation

Moderator	CGN variance	CGN-SO covariance	SO variance
	Estimate (95% CI)	Estimate (95% CI)	Estimate (95% CI)
*CGN (Model 1)*^a^			
*β*_A_		0.03 (− 0.14, 0.20)	0.05 (− 0.30, 0.27)
*β*_E_		0.01 (− 0.18, 0.17)	− **0.38 (**− **0.52,** − **0.001)**
*ELA (Model 2)*^b^			
*β*_A_	− 0.02 (− 0.12, 0.09)	0.02 (− 0.15, 0.24)	− 0.13 (− 0.33, 0.33)
*β*_E_	0.03 (− 0.02, 0.10)	0.09 (− 0.07, 0.22)	− 0.03 (− 0.49, 0.18)

Bold parameters indicate statistically signficant parameter estimates

*ELA* Early-life adversities, *CGN* Childhood gender nonconformity, *SO* Sexual orientation, *β*_A_, *β*_E_ Moderation coefficient for additive genetic and non-shared environmental components, respectively

^a^In this model, the moderator (CGN) was included as a dependent variable, and all unmoderated and moderated C parameters were dropped. Degrees of freedom = 6412, − 2LL = 16,990.52, AIC = 4166.52

^b^In this model, the moderator (ELA) was included as a dependent variable, and all unmoderated and moderation C parameters were dropped. Degrees of freedom = 9610, − 2LL = 19,239.29, AIC = 19.29142

In the second genetic model (ELA as moderator), ***A*** and ***E*** influences on the variance of CGN were not significantly moderated by ELA (*β*_A_ =  − 0.02; 95% CI − 0.12, 0.09; *β*_E_ = 0.03; 95% CI − 0.02, 0.10, Table 5, Fig. 3). Similarly, ***A*** and ***E*** influences on the covariance between childhood gender nonconformity and sexual orientation (*β*_A_ = 0.02; 95% CI − 0.15, 0.24; *β*_E_ = 0.09; 95% CI − 0.07, 0.22, Table 5, Fig. 3) and on the variance of sexual orientation (*β*_A_ =  − 0.13; 95% CI − 0.33, 0.33; *β*_E_ =  − 0.03; 95% CI − 0.49, 0.18, Table 5, Fig. 3) were not significantly moderated by ELA.

## Discussion

### Phenotypic and Etiological Correlations

Consistent with existing evidence (Li et al., 2017) and previous analyses using the present data (Alanko et al., 2010), the present study found significant positive phenotypic correlations between sexual orientation (SO) and childhood gender nonconformity (CGN). The present study went beyond the earlier analysis by Alanko et al. (2010) by further demonstrating a positive phenotypic association between sexual orientation and early-life adversities (ELA) as has been shown in non-twin studies (e.g., Baams, 2018). The variances in SO, CGN and ELA were each significantly influenced by additive genetic and non-shared environmental influences as have been previously reported (Burri et al., 2015; Sartor et al., 2012). The genetic correlation between CGN and SO is consistent with previous findings (Bailey et al., 2000; Burri et al., 2015) and indicates common genetic influences. The correlated individual-specific environmental factors may reflect neurohormonal factors which jointly affect sexual orientation and childhood gender nonconformity (Bao & Swaab, 2011; Reinisch et al., 2017), however, this correlation may also be inflated by correlated measurement error.

The positive phenotypic correlation between CGN and ELA may be mediated by poorer parent–child relationships, whereby CGN results in poor relationships with parents (Alanko et al., 2008) which can facilitate emotional and physical abuse and neglect (D’Augelli et al., 2006; Rikhye et al., 2008). Poor parent–child relationships have also been shown to be causally related to CGN (Alanko et al., 2011), which raises the possibility of reverse causation. This is consistent with the counter-normative model in which disruptive family settings may facilitate non-normative (nonconforming) gender expression (Rind, 2013). Alternatively, the observed significant genetic and individual-specific correlations between CGN and ELA found in this study may indicate pleiotropic effects of shared etiological influences or transmission of these influences through causal paths (Wedow et al., 2018). Although the significant phenotypic correlation between ELA and SO is consistent with a previous finding (Baams, 2018), the attenuation of this relationship after including CGN in the model is also consistent with a previous finding (Xu et al., 2020) and suggests that the association between early-life adversities and sexual orientation is confounded by CGN.

#### Moderation by Childhood Gender Nonconformity and Early-Life Adversity

The variance of SO and the relationship between CGN and SO were significantly moderated by CGN while ELA only significantly moderated the variance of CGN. Of these relationships, only the moderation of SO variance was shown to be due to moderation of individual-specific environmental influences which significantly decreased as CGN increased.

#### Moderation by Childhood Gender Nonconformity

The variance of SO was lowest at the highest levels of CGN. Taken together with the positive association between SO and CGN found in the present and previous studies (Bailey & Zucker, 1995; Li et al., 2017); this finding indicates the importance of CGN in understanding the development of sexual orientation. Specifically, our finding can be interpreted to indicate that the “noise” or random influences around the etiological mechanisms of sexual orientation diminish as childhood gender nonconformity increases. This interpretation is supported by the complete attenuation of individual-specific environmental influences (which include stochastic processes and measurement error; Neale & Cardon, 1992; Rijsdijk & Sham, 2002) at the highest levels of CGN. In contrast, the change in genetic influences on sexual orientation by CGN was relatively smaller and not statistically significant. Thus, CGN—or other factors indexed by it (such as prenatal neurohormonal influences)—may facilitate the expression of sexual orientation by diminishing regulatory individual-specific influences such as epigenetic processes (Rice et al., 2013); however, this possibility needs to be specifically investigated.

The stronger phenotypic relationship between CGN and SO at higher levels of CGN is consistent with previous reports (Bailey & Zucker, 1995; Li et al., 2017) but the present study could not establish significance of the underlying genetic and environmental influences of this moderation effect. This may reflect low power of the present study; however, the uniform increase in component influences on this relationship with CGN can suggest that the same genetic and individual-specific processes underlie the association between CGN and SO across all levels of CGN.

#### Moderation by Early-Life Adversity

The increasing phenotypic variance of CGN with increasing early-life adversities suggests that despite the known positive association between childhood gender nonconformity and negative experiences during childhood from previous research (D’Augelli et al., 2006); other factors are likely to be as important as or even more so than early-life adversities in understanding childhood gender nonconformity. While evidence from animal models suggests that stress in the neonatal period can influence gendered behavior (Toufexis et al., 2014), it is also possible that increasing childhood gender nonconformity elicits more negative reactions from parents and others in the child’s environment (D’Augelli et al., 2006). However, such negative reactions may not be uniformly elicited from others and this inconsistency may explain the explain the increased variance of CGN observed at high levels of ELA in this study and is consistent with increasing ***E*** influences on CGN variance at high higher levels of ELA although this moderation effect was not statistically significant in the present study.

In contrast to moderation by CGN, the variance of SO did not significantly vary by the level of early-life adversities. Considering that the relationship between early-life adversities and sexual orientation is confounded by CGN, it is possible that early-life adversities are not independently associated with sexual orientation. Similarly, moderation of the variance in sexual orientation by ELA could not be resolved into moderation of the genetic and environmental influences. Although this may reflect low power, it is also possible that ELA is not independently associated with the etiological influences on sexual orientation.

Regarding the relationship between CGN and SO, this did not significantly vary across different levels of ELA. These findings suggest that the phenotypic or etiological correlation between SO and CGN are largely independent of ELA. However, the large confidence intervals also raise the possibility of low power and highlight the need for larger samples in future studies.

#### Relevance to the Minority Stress Model

Our findings may be extended to further explain the mental health disparities in non-heterosexual compared to heterosexual individuals which is typically attributed to minority stress (Meyer, 2003). We highlight the importance of childhood gender nonconformity in understanding the mental health disparities among non-heterosexual individuals. While childhood gender nonconformity appears to be associated with diminished overall variance and individual-specific environmental influences on non-heterosexuality, it also facilitates exposure to adverse childhood experiences (Alanko et al., 2011; D’Augelli et al., 2006). Childhood gender nonconformity may thus differentially increase the likelihood of psychopathology among non-heterosexual individuals during development (Friedman et al., 2006; Jones et al., 2017; Oginni et al., 2019, 2022) possibly through epigenetic processes (Heim & Binder, 2012). Given the associations between adult psychopathology, and childhood adversity (Heim & Binder, 2012), childhood psychopathology (Zarrella et al., 2017) and sexual minority stress (Meyer, 2003); children who are extremely gender nonconforming may be specifically targeted for supportive interventions to minimize their risk for victimization and increased risk for current and later psychopathology. Such interventions may include education and counselling of their parents and guardians, early identification and treatment of childhood psychopathology and resilience training; however, the utility of these need to be specifically investigated.

### Limitations

This is the first study to investigate moderation of genetic and environmental influences on sexual orientation and a major strength is the assessment of variables of interest using standardized instruments with good psychometric properties. However, in interpreting our findings, the following limitations need to be considered. The assessments of CGN and ELA were retrospective and may be subject to recall bias, however, our findings are consistent with longitudinal evidence that gender nonconformity and adversities in childhood are significantly higher in non-heterosexual compared to heterosexual individuals (Andersen & Blosnich, 2013; Li et al., 2017; Xu et al., 2020). Relatedly, our assessment of ELA included the period up to 18 years, which is later than the age at which many individuals recognize their non-heterosexuality (Dunlap, 2016). Thus, any influences of ELA on the etiological mechanisms of SO should have occurred earlier. Our assessment may therefore have included participants who experienced adversities outside the relevant developmental period, in which case the adversities could be secondary to CGN or non-heterosexuality rather than vice versa. However, there is evidence that exposure to adversity in middle childhood and adolescence is more likely to occur as a continuation from early childhood rather than as new exposures (Dierkhising et al., 2019). Furthermore, novel genetic and environment influences on individual trait differences have been shown to emerge throughout development (up to 18 years) and only become stable in early adulthood (Kendler et al., 2008; Nivard et al., 2015) but this has not been specifically demonstrated for sexual orientation.

A recognized limitation of studies investigating sensitive subject matters such as sexual orientation is low response rate (Barth et al., 2016). This may result in selection bias such that individuals who are more comfortable with their sexuality may be over-represented in the study sample and limit the generalizability of the study findings. However, the response rate in the present study is comparable to those from population-based studies using similar methods (Guo et al., 2016), and the characteristics of the present study’s sample are comparable to those of other representative, population-based Finnish samples (as previously described by Alanko et al., 2010). Concordance-dependent ascertainment bias in which a disproportionately larger number of concordant monozygotic relative to dizygotic twins are recruited may result in an overestimation of additive genetic influences on sexual orientation (Bailey et al., 1993). However, the proportion of non-heterosexual participants was comparable among monozygotic and dizygotic twins in the present study.

Another concern about the generalizability of our findings is that the assessment of the direct and moderated influence of the variance components is specific to the time and population in which the study was carried out. Our findings need to be replicated using independent twin datasets and as the wide confidence intervals indicate the need for larger sample sizes, future datasets could be derived by combining multiple twin cohorts. Given evidence for sex differences in the relationship between childhood gender nonconformity and sexual orientation (Li et al., 2017; Watts et al., 2018), it would have been beneficial to investigate sex differences in the present analyses. However, the number of concordant male pairs was small, and this precluded a reliable estimation of the variance component influences. A larger sample size could overcome this limitation and facilitate the investigation of sex differences in these relationships in future studies. A larger sample size would also facilitate incorporating more sexual orientation categories which can better reflect the spectrum of sexual orientation (Savin-Williams & Vrangalova, 2013) and increase power in twin data analyses involving ordinal variables (Neale et al., 1994).

Finally, while the twin method estimates the overall magnitude of genetic and environmental etiological influences, it does not identify these specific factors. Thus, while suggested explanations of our findings are based on existing theory and empirical research, they remain speculative. Though we interpret the *E* as indicating individual-specific environmental effects, it is important to bear in mind that this component incorporates measurement error which may lead to false conclusions about unique environmental influences.

### Conclusion and Implications

In view of these limitations, positive findings from statistical genetic methods such as ours may at best be taken to suggest possible etiological mechanisms which need to be explored further using alternative designs to identify specific genetic and non-genetic processes. This is illustrated by the relatively longer-standing evidence of genetic influences on sexual orientation from twin studies which were only recently confirmed by a genome-wide association study (Ganna et al., 2019).

Taken together, the moderation of the variance in sexual orientation by CGN suggests that in addition to its direct associations with SO; CGN may influence individual differences in SO by reducing individual-specific environmental influences (including error) on SO. As CGN was assessed over a relatively wide developmental window, our findings raise the possibility that etiological influences on sexual orientation are dynamic rather than static and may be moderated at critical periods; however, this needs to be empirically tested (e.g., using longitudinal designs).

Our findings thus extend the knowledge about the mechanisms of sexual orientation by suggesting alternative processes which can be further investigated. This knowledge may help non-heterosexual individuals come to a better understanding and acceptance of themselves. Improved understanding has been shown to be associated with less self-stigma and better psychological wellbeing among non-heterosexual individuals (Morandini et al., 2015, 2017). More indirectly, a clearer understanding of the biological mechanisms of sexual orientation may facilitate policy change to reduce sexuality-related discrimination especially in highly homophobic regions such as in many low- and middle-income countries (Bailey et al., 2016). Considering that CGN is associated with psychological distress in childhood (Jones et al., 2017) and adulthood (Oginni et al., 2019), a clearer understanding of its relationship with SO can shed also more light on the association between SO and psychological distress.

### Supplementary Information

Below is the link to the electronic supplementary material.

Supplementary file1 (DOCX 90 KB)
