# Supplementary material for: Genetic and Environmental Influences on Sexual Orientation: Moderation by Childhood Gender Nonconformity and Early-Life Adversity
Source: Arch Sex Behav. 2023 Dec 28;53(5):1763–76. doi: 10.1007/s10508-023-02761-w (PMC11106125; doi:10.1007/s10508-023-02761-w)
Supplement: Supplementary file 1 — Supplementary file1 (DOCX 90 KB) [file 10508_2023_2761_MOESM1_ESM.docx]

**Table S1: Distribution of sexual orientation.**

|  | **(n = 3558)** | |
| --- | --- | --- |
| **Variables** | **Missingness** | |
|  | **n** | **%** |
| Age | 0 | 0.00 |
| Sex | 0 | 0.00 |
| Same-sex attraction (number) | 53 | 1.50 |
| Same-sex attraction (frequency) | 58 | 1.60 |
| Same-sex behaviour (number) | 62 | 1.70 |
| Same-sex behaviour (frequency) | 61 | 1.70 |
| CGN – Item 1 | 25 | 0.70 |
| CGN – Item 2 | 23 | 0.60 |
| CGN – Item 3 | 33 | 0.90 |
| CGN – Item 4 | 21 | 0.60 |
| CGN – Item 5 | 35 | 1.00 |
| CGN – Item 6 | 32 | 0.90 |
| CGN – Item 7 | 28 | 0.80 |
| CGN – Item 8 | 42 | 1.20 |
| CGN – Item 9 | 22 | 0.60 |
| CGN – Item 10 | 29 | 0.80 |
| CGN – Item 11 | 43 | 1.20 |
| CGN – Item 12 | 65 | 1.80 |
| CGN – Item 13 | 53 | 1.50 |
| ELA – Item 1 | 17 | 0.50 |
| ELA – Item 2 | 20 | 0.60 |
| ELA – Item 3 | 24 | 0.70 |
| ELA – Item 4 | 18 | 0.50 |
| ELA – Item 5 | 20 | 0.60 |
| ELA – Item 6 | 24 | 0.70 |
| ELA – Item 7 | 14 | 0.40 |
| ELA – Item 8 | 17 | 0.50 |
| ELA – Item 9 | 20 | 0.60 |
| ELA – Item 10 | 21 | 0.60 |
| ELA – Item 11 | 16 | 0.40 |
| ELA – Item 12 | 19 | 0.50 |
| ELA – Item 13 | 20 | 0.60 |
| ELA – Item 14 | 19 | 0.50 |
| ELA – Item 15 | 24 | 0.70 |
| ELA – Item 16 | 16 | 0.40 |
| ELA – Item 17 | 32 | 0.90 |
| ELA – Item 18 | 23 | 0.60 |
| ELA – Item 19 | 18 | 0.50 |
| ELA – Item 20 | 28 | 0.80 |
| ELA – Item 21 | 15 | 0.40 |
| ELA – Item 22 | 15 | 0.40 |
| ELA – Item 23 | 20 | 0.60 |
| ELA – Item 24 | 17 | 0.50 |
| ELA – Item 25 | 15 | 0.40 |

**CGN** – Childhood Gender Nonconformity, **ELA** – Early-Life Adversity

**Table S2: Within-person and cross-twin correlations of study variables with 95% confidence intervals per sex and zygosity groups.**

|  | **Male** |  |  | **Female** |  |  |
| --- | --- | --- | --- | --- | --- | --- |
|  | **ELA (1)** | **CGN (2)** | **SO (3)** | **ELA (1)** | **CGN (2)** | **SO (3)** |
| **Within person** |  |  |  |  |  |  |
| **(1)** | 1 |  |  | 1 |  |  |
| **(2)** | 0.13  (0.07, 0.19) | 1 |  | 0.15  (0.11, 0.20) | 1 |  |
| **(3)** | 0.11  (0.00, 0.22) | 0.43  (0.35, 0.51) | 1 | 0.27  (0.19, 0.34) | 0.21  (0.13, 0.29) | 1 |
|  |  |  |  |  |  |  |
| **Cross-twin** |  |  |  |  |  |  |
| **Monozygotic** |  |  |  |  |  |  |
| **(1)** | 0.45  (0.27, 0.58) |  |  | 0.68  (0.62, 0.74) |  |  |
| **(2)** | 0.09  (-0.02, 0.21) | 0.44  (0.26, 0.57) |  | 0.11  (0.04, 0.17) | 0.51  (0.42, 0.59) |  |
| **(3)** | -0.01  (-0.26, 0.24) | 0.14  (-0.10, 0.36) | -0.16  (-0.85, 0.64) | 0.22  (0.09, 0.33) | 0.23  (0.10, 0.36) | 0.67  (0.36, 0.86) |
|  |  |  |  |  |  |  |
| **Dizygotic** |  |  |  |  |  |  |
| **(1)** | 0.28  (0.10, 0.43) |  |  | 0.47  (0.37, 0.55) |  |  |
| **(2)** | 0.10  (-0.04, 0.23) | 0.24  (0.00, 0.42) |  | 0.09  (0.01, 0.17) | 0.08  (-0.04, 0.20) |  |
| **(3)** | 0.31  (0.05, 0.54) | 0.29  (-0.03, 0.52) | 0.62  (-0.88, 0.92) | 0.03  (-0.12, 0.17) | -0.09  (-0.25, 0.07) | 0.12  (-0.28, 0.48) |
|  |  |  |  |  |  |  |
| **Dizygotic opposite-sex** |  |  |  |  |  |  |
| **(1)** | 0.17  (0.03, 0.30) |  |  |  |  |  |
| **(2)** | 0.01  (-0.09, 0.11) | -0.13  (-0.27, 0.01) |  |  |  |  |
| **(3)** | 0.15  (-0.03, 0.32) | 0.02  (-0.18, 0.21) | -0.07  (-0.59, 0.46) |  |  |  |

**ELA:** Early-life adversities, **CGN:** Childhood gender nonconformity, **SO:** Sexual orientation.

**Figure S1: Moderation of the phenotypic variance-covariance relationship between childhood gender nonconformity (CGN) and sexual orientation (SO) by CGN (*a* and *b*) and early-life adversity (ELA, *c, d* and *e*).** Changes in unstandardized SO-CGN variances and covariance are plotted as functions of moderators (CGN and ELA).

|  |  | | |
| --- | --- | --- | --- |
| **Moderator** | **Variance of**  **childhood gender nonconformity** | **Covariance of sexual orientation**  **and childhood gender nonconformity** | **Variance of**  **sexual orientation** |
| **Childhood Gender Nonconformity** |  | 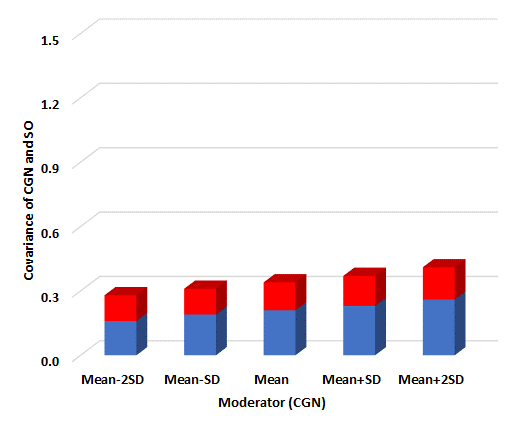 | 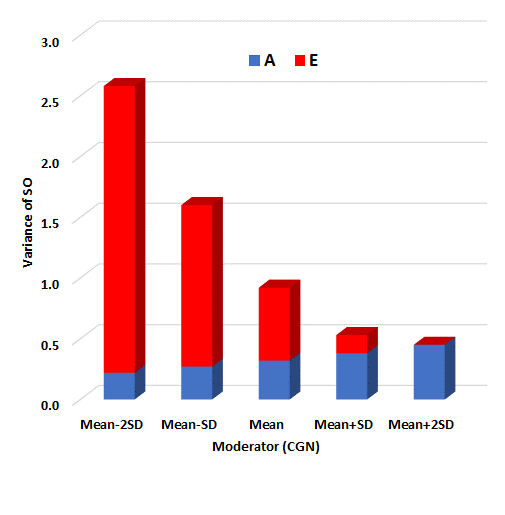 |
| **Early-Life Adversities** | 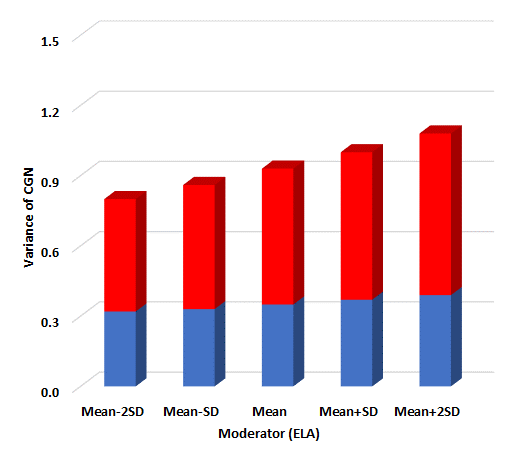 | 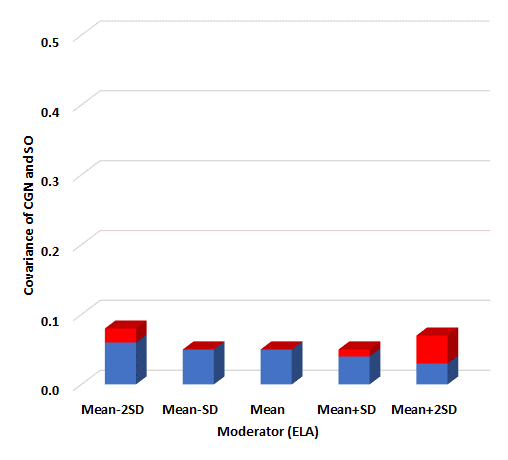 | 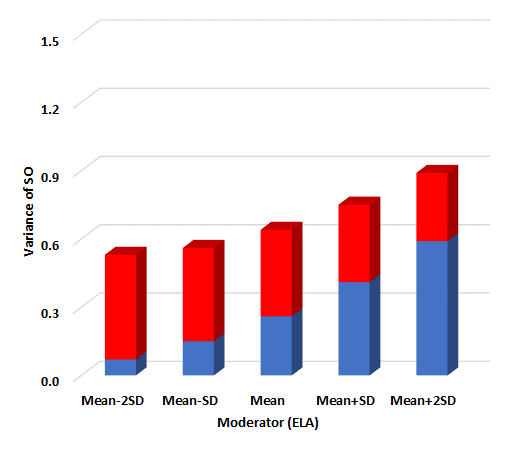 |

**Figure S2: Moderation of covariance between childhood gender nonconformity (CGN) and sexual orientation (SO) and variance in SO by CGN (*a* and *b* respectively) and early-life adversity (ELA, *c* and *d* respectively).** Changes in unstandardized SO-CGN covariance and SO variance are plotted as functions of moderators (CGN and ELA). **A,** and **E:** Additive genetic and Non-shared environmental components respectively.
